# Isolation and Characterization of Functional Tripartite Group II Introns Using a Tn5-Based Genetic Screen

**DOI:** 10.1371/journal.pone.0041589

**Published:** 2012-08-02

**Authors:** Christine Ritlop, Caroline Monat, Benoit Cousineau

**Affiliations:** Department of Microbiology and Immunology, McGill University, Montréal, Québec, Canada; International Centre for Genetic Engineering and Biotechnology, Italy

## Abstract

**Background:**

Group II introns are RNA enzymes that splice themselves from pre-mRNA transcripts. Most bacterial group II introns harbour an open reading frame (ORF), coding for a protein with reverse transcriptase, maturase and occasionally DNA binding and endonuclease activities. Some ORF-containing group II introns were shown to be mobile retroelements that invade new DNA target sites. From an evolutionary perspective, group II introns are hypothesized to be the ancestors of the spliceosome-dependent nuclear introns and the small nuclear RNAs (snRNAs – U1, U2, U4, U5 and U6) that are important functional elements of the spliceosome machinery. The ability of some group II introns fragmented in two or three pieces to assemble and undergo splicing in *trans* supports the theory that spliceosomal snRNAs evolved from portions of group II introns.

**Methodology/Principal Findings:**

We used a transposon-based genetic screen to explore the ability of the Ll.LtrB group II intron from the Gram-positive bacterium *Lactococcus lactis* to be fragmented into three pieces *in vivo*. *Trans*-splicing tripartite variants of Ll.LtrB were selected using a highly efficient and sensitive *trans*-splicing/conjugation screen. We report that numerous fragmentation sites located throughout Ll.LtrB support tripartite *trans*-splicing, showing that this intron is remarkably tolerant to fragmentation.

**Conclusions/Significance:**

This work unveils the great versatility of group II intron fragments to assemble and accurately *trans*-splice their flanking exons *in vivo*. The selected introns represent the first evidence of functional tripartite group II introns in bacteria and provide experimental support for the proposed evolutionary relationship between group II introns and snRNAs.

## Introduction

Self-splicing group II introns were initially discovered interrupting various mitochondrial and chloroplastic genes of higher plants and algae, as well as mitochondrial genes of lower eukaryotes [1]. While uncommon in Archaea, group II introns were uncovered in both Gram-positive and Gram-negative bacteria, where they are generally present in one to a few copies per genome [1]. In contrast, group II introns do not interrupt nuclear encoded genes of eukaryotes. Instead, nuclear genes are interrupted by a different type of intervening sequence, spliceosomal introns.

**Figure 1:**
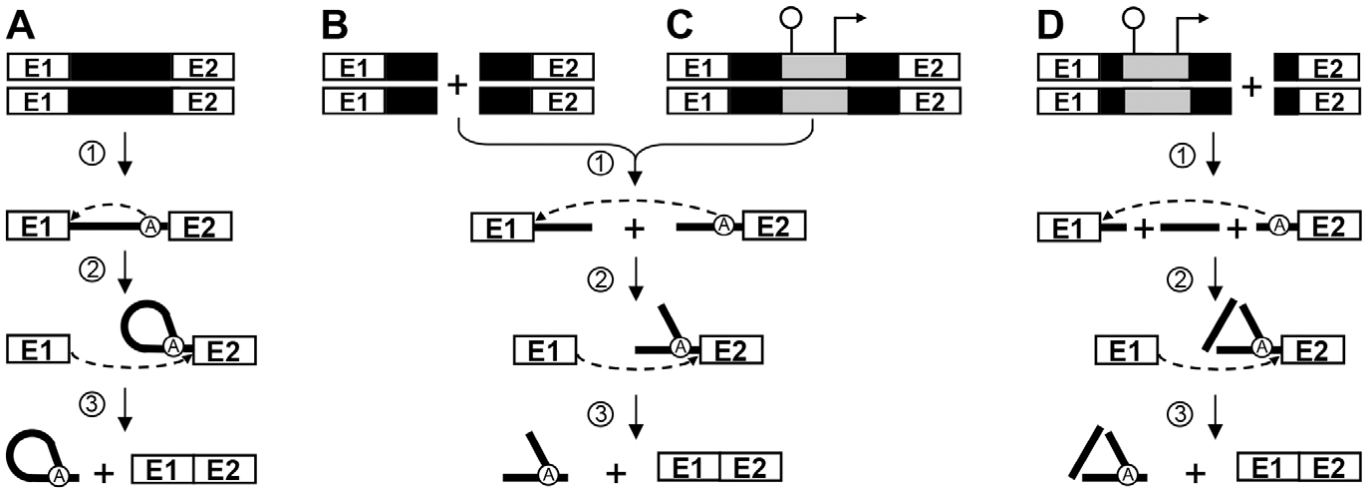
Group II intron splicing pathways. (*A*) *Cis*-splicing. Following transcription of the interrupted gene (step *1*), the 2′-OH residue of the branch-point nucleotide (A) performs a nucleophilic attack at the exon 1-intron junction (step *2*). The liberated 3′-OH at the end of exon 1 then initiates the second nucleophilic attack at the intron-exon 2 junction (step *3*), ligating the two exons and releasing the intron in the form of a lariat. (*B*) *Trans*-splicing of bipartite group II introns. Fragmented group II introns that are expressed from separate loci (step *1*) can assemble and splice *in trans* to ligate their flanking exons and release a Y-branched intron molecule (steps *2* and *3*). (*C*) *Trans*-splicing of Tn5-interrupted bipartite group II introns. Insertion of a Tn5 transposon, harboring a transcriptional terminator followed by a constitutive promoter within the intron, results in the expression of the gene as two separate transcripts (step *1*). These two transcripts assemble and undergo *trans*-splicing leading to the ligation of their flanking exons and release a Y-branched intron molecule (steps *2* and *3*). (*D*) *Trans*-splicing of Tn5-interrupted tripartite group II introns. Insertion of a Tn5 transposon within the first fragment of a bipartite *trans*-splicing intron results in the expression of the gene as three separate transcripts (step *1*). The three intron fragments assemble and splice in *trans* ligating the flanking exons (steps *2* and *3*). The Tn5 transposon can also be used to fragment the second piece of a *trans*-splicing bipartite intron. Group II intron, black box (DNA) and black line (RNA); branch point, circled A; Exon 1 and 2, E1 and E2; Tn5 transposon, grey box; *pepN* transcriptional terminator, schematic stem-loop; P_23_ constitutive promoter, right-angle arrow.

Despite a lack of sequence similarity, group II introns fold into a conserved secondary structure composed of six domains (DI-DVI) radiating from a central wheel [1]–[3]. DI, the largest of the six domains and the first to be transcribed, serves as a scaffold to dock the remaining domains (DII-DVI), and is therefore involved in several important long-range tertiary interactions [4]–[7]. The lower stem of DII recruits DIII to the catalytic core, with DIII acting as a catalytic effector [8]. DIV often contains an open reading frame (ORF) coding for an intron-encoded protein (IEP) with reverse transcriptase, maturase, and occasionally DNA binding, and endonuclease activities. This domain of variable size is not essential for splicing [9], and accordingly protrudes from the catalytic core of the active tertiary structure [5], [6]. Some ORF-containing group II introns were shown to be mobile retroelements invading identical or ectopic DNA target sites through retrohoming or retrotransposition, respectively [1]. DV is the catalytic domain of these ribozymes, and consequently exhibits the most sequence conservation among group II introns [2]. Its interaction with DI forms the minimal catalytic core required for self-splicing [4]. DVI carries the bulged adenosine (A) residue, also termed the branch point, responsible for the first nucleophilic attack of the splicing pathway (Fig. 1) [1]. In addition to local interactions, a series of long-range tertiary contacts between the domains contributes to the three-dimensional folding of group II introns, that must take place to enable splicing [4]–[7].

Group II introns are RNA enzymes that splice autocatalytically both *in vivo* and *in vitro*. *In vivo* however, folding of these large ribozymes into their catalytically active structure depends on the maturase activity of their IEP or free-standing maturases. Group II introns self-splice from pre-mRNA transcripts through two consecutive transesterification reactions (Fig. 1A) [1]. Following transcription of the interrupted gene (Fig. 1A, step 1), the first nucleophilic attack at the exon 1-intron junction is initiated by the 2′-OH residue of a bulged adenosine (A) residue located near the 3′ end of the intron (Fig. 1A, step 2). The liberated 3′-OH of exon 1 then initiates the second transesterification reaction at the intron-exon 2 junction (Fig. 1A, step 3), which concurrently leads to the ligation of the two flanking exons and the release of the intron RNA in the form of a lariat.

From an evolutionary perspective, group II introns are considered to be the ancestors of eukaryotic nuclear introns and the small nuclear RNAs (snRNAs – U1, U2, U4, U5, U6) that constitute the heart of the nuclear intron splicing machinery called the spliceosome [2], [9]–[17]. Nuclear introns follow the same splicing pathway as group II introns and are also released as branched RNA lariats (Fig. 1A) even though they are not autocatalytic and removed by the spliceosome. This functional relationship strongly suggests that group II and nuclear introns share a common ancestor [10], [12], [13]. Similar signature sequences at the exon-intron boundaries between group II and nuclear introns also support this evolutionary link [13].

The snRNAs of the eukaryotic spliceosome are hypothesized to have originated from the fragmentation of an ancestral group II intron into *trans*-acting RNAs [11]. In accordance, substantial evidence indicates that the splicing reaction achieved by the spliceosome is fundamentally RNA-mediated, strengthening the argument that this large splicing machinery evolved from a primordial ribozyme [18]. Further evidence supporting this theory includes various functional and/or structural similarities between some snRNAs and portions of group II introns [17]. In addition, some group II introns are found to be fragmented as a result of genome rearrangements in organelles [19]–[20]. Remarkably, these intron fragments have the capacity to assemble and accurately splice their flanking exons in *trans* (Fig. 1B), strengthening the argument that the snRNAs of the spliceosome were generated from group II intron fragments [11], [16], [17]. Most *trans*-splicing group II introns are bipartite, with their fragmentation sites occurring within DIV, either upstream or downstream of the ORF, or at the tip of DIII. Two tripartite introns fragmented within DI and DIV were also identified interrupting the chloroplast *psaA* gene of *Chlamydomonas reinhardtii* [21] and the mitochondrial *nad5* gene of *Oenothera berteriana* [22]. These naturally occurring bipartite and tripartite introns provide a glimpse of the intermediate steps that may have occurred in the evolutionary transition from group II intron fragments to spliceosomal snRNAs [11], [21].

The Ll.LtrB intron from the Gram-positive bacterium *Lactococcus lactis* is a well-characterized bacterial group II intron [1]. This 2.5 kb intron carries an ORF in DIV coding for LtrA, a 599 amino acid protein with reverse transcriptase, maturase, DNA binding, and endonuclease activities [23]. LtrA is an essential splicing co-factor for Ll.LtrB that promotes the catalytically active tertiary conformation of the ribozyme [23]. Ll.LtrB is found on three *L. lactis* conjugative elements, the pAH90 plasmid [24] and two highly similar elements, the pRS01 plasmid [25] and the chromosomal sex factor (SF) [26]. In all cases, Ll.LtrB interrupts *ltrB*, a gene coding for relaxase (LtrB). Relaxase initiates conjugation by nicking the conjugative element at the origin of transfer (*oriT*) and delivers the DNA to recipient cells [27]. Given that this enzyme is essential to trigger conjugation, splicing of Ll.LtrB from its pre-mRNA transcript is necessary for LtrB production and subsequent DNA transfer. Therefore, Ll.LtrB splicing controls the conjugation efficiency of its host elements [16], [17], [25], [26], [28]. We previously developed a sensitive *trans*-splicing/conjugation assay in *L. lactis*, showing that Ll.LtrB is able to splice in *trans* when fragmented at natural group II intron fragmentation sites [16]. Using a transposon-based genetic screen, we then demonstrated that Ll.LtrB efficiently *trans*-splices when fragmented at various locations throughout its structure, revealing its *trans*-splicing versatility [17].

Here, we took advantage of our previously engineered Tn5-based genetic screen to explore sites throughout Ll.LtrB that would tolerate fragmentation into three pieces. Tripartite *trans*-splicing Ll.LtrB variants were randomly generated and selected using an *in vivo trans*-splicing/conjugation assay. We discovered that Ll.LtrB can assemble and accurately *trans*-splice when fragmented into three pieces at many locations throughout the majority of its structure. Moreover, some of the Ll.LtrB tripartite variants studied were found to *trans*-splice only 11- to 35-fold less efficiently than their bipartite counterparts. This is the first demonstration of the tripartite *trans*-splicing ability of Ll.LtrB, as well as the tripartite *trans*-splicing versatility of a bacterial group II intron. This study further illustrates the great plasticity of group II intron to fragmentation and supports the theory suggesting that spliceosomal snRNAs were derived from group II intron fragments.

## Results

### *Trans*-splicing/conjugation Assay in *L. lactis*

We previously established two very sensitive group II intron *trans*-splicing/conjugation assays in *L. lactis*. In one assay, *trans*-splicing of Ll.LtrB fragmented in two pieces at natural group II intron fragmentation sites was monitored by conjugation of a relaxase-deficient chromosomal sex factor (SF) (10^7^-fold detection range) [16]. The second assay involved a transposon-based genetic screen, where we selected and characterized proficient Ll.LtrB *trans*-splicers fragmented at various positions throughout the intron by the conjugation of their carrying pLE12 mobilizable plasmid (10^4^-fold detection range) [17].

**Figure 2:**
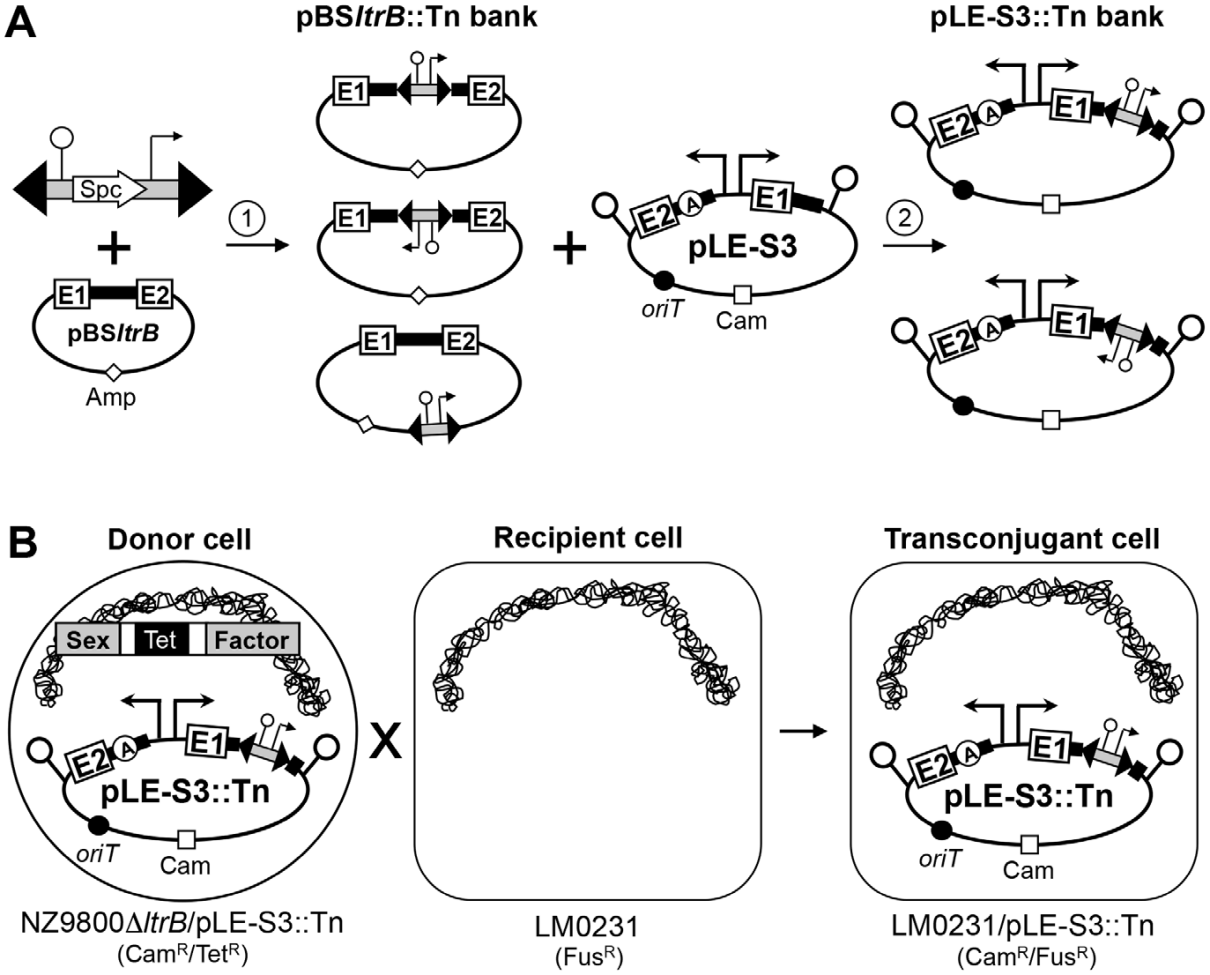
*In vivo* functional assay to select for tripartite *trans*-splicing introns. (*A*) Generation of a saturated Tn5 insertion bank. The Tn5 transposon was mixed with the target plasmid (pBS*ltrB*) in presence of the Tn5 transposase (step *1*). This *in vitro* reaction created a bank of Tn5 insertions within the target plasmid (pBS*ltrB*::Tn bank) consisting of unique insertions between every nucleotide [17]. To generate the pLE-S3::Tn bank, bearing Tn5 insertions exclusively in a portion of the first fragment of the S3 Ll.LtrB bipartite intron, the corresponding section was excised (PvuI/BsaI) from the pBS*ltrB*::Tn bank and transferred to pLE-S3 (step *2*). The pLE-S4::Tn (BsrGI/AatII) and pLE-S10::Tn (EagI/BsaI) banks bearing Tn5 insertions exclusively in a portion of the second intron fragment were generated the same way (*B*) *Trans*-splicing/conjugation assay. The pLE-S3::Tn bank is first introduced in the NZ9800Δ*ltrB* donor strain and mated with the LM0231 recipient strain. This assay selects for plasmids harboring tripartite Ll.LtrB *trans*-splicing variants. Every donor cell contains a different plasmid, only the pLE-S3::Tn variants that harbor a *trans*-splicing tripartite Ll.LtrB intron produce relaxase and are transferred by conjugation. The same procedure was followed with the pLE-S4::Tn and pLE-S10::Tn banks. Origin of conjugative transfer (*oriT*), black circle.

**Figure 3:**
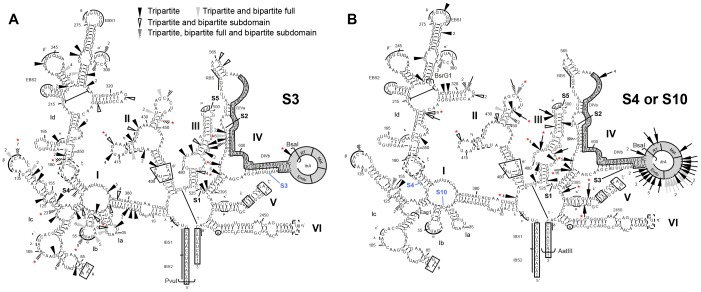
Selected Tn5 insertion sites supporting Ll.LtrB tripartite *trans*-splicing. The Tn5 tripartite screen was performed independently on the first fragment of S3 (PvuI/BsaI) (*A*) and the second fragment of S4 (BsrGI/AatII) and S10 (EagI/BsaI) (*B*). Arrowheads (S3, S10) and complete arrows (S4) represent the position of tripartite fragmentation sites, with their frequencies indicated numerically. Fragmentation sites are represented by different arrowheads/arrows: uniquely uncovered in the tripartite screens, black; previously observed in bipartite screens of the full-length intron, gray; also observed in previous subdomain screens, open; observed in all previous screens, checkered [17]. Red asterisks highlight tripartite fragmentation sites chosen for further investigation. The fragmentation points that were previously engineered to mimic natural *trans*-splicing group II introns are denoted S1-S5 [16] and S10. Insertion of the Tn5 transposon creates a 9-nt direct repeat of the target sequence [30]: the end of the first fragment is indicated by the arrowheads/arrows, while the second fragment starts 9 nt upstream. Exon binding sequences 1 and 2, EBS1 and 2; intron binding sequences 1 and 2, IBS1 and 2; Exons, open boxes; *ltrA*, grey box; LtrA reverse transcriptase domain, RT; LtrA maturase domain, Mat; LtrA endonuclease domain, Endo.

Here, we developed another assay to select and monitor Ll.LtrB *trans*-splicing efficiency in three pieces by the conjugative transfer of its carrying pLE1 mobilizable plasmid (Fig. 2). pLE1 was engineered to contain the origin of transfer (*oriT*) of the *L. lactis* SF so that it could be mobilized upon relaxase production from tripartite *trans*-spliced Ll.LtrB transcripts (Fig. 2B). The strategy was to subject one of the two intron pieces from specific bipartite introns to random Tn5 fragmentation (Fig. 2A) and select for functional tripartite introns by conjugation (Fig. 2B). Three different bipartite introns were subjected to such a screen (Fig. 3, S3, S4, S10). In each case, the *ltrB* gene was cloned as two segments, *ltrB*Exon1–5′-intron and 3′-intron–*ltrB*Exon2; both of which were placed under control of divergent *L. lactis* constitutive promoters (P_23_) (Fig. 2A). Previous work had identified S3 and S4 as efficient bipartite *trans*-splicers, where chromosomal SF transfer has been shown to occur at efficiencies of 1.2×10^−2^ and 4.5×10^−4^ respectively, compared to 5.8×10^−2^ for Ll.LtrB *cis*-splicing [16]. The third fragmentation site at S10 mimics a previously selected bipartite intron supporting the transfer of its carrying plasmid almost as efficiently as the *cis*-splicing intron, 2.6×10^−5^ *vs* 3.2×10^−5^ respectively [17]. It is important to note that the difference in conjugation efficiency achieved by the *cis*-splicing intron depends on the conjugation assay used: 3.2×10^−5^ for plasmid transfer *vs* 5.8×10^−2^ for chromosomal SF transfer.

NZ9800Δ*ltrB*::*tet* was used as the donor strain for plasmid conjugation (Fig. 2B). This *L. lactis* strain bears a defective relaxase gene within the chromosomal SF and therefore does not permit SF transfer in the absence of LtrB [16]. The pLE-based plasmids harboring bipartite introns (pLE-S3, pLE-S4, pLE-S10) transferred by conjugation from NZ9800Δ*ltrB* to the LM0231 recipient strain with good efficiencies in the 10^−5^ to 10^−6^ range compared to 10^−9^ for the empty vector (Table 1). These results indicated that a 10^3^- to 10^4^-fold detection range was available to screen for variants of Ll.LtrB that can *trans*-splice when further fragmented into three pieces.

**Table 1:** Conjugation efficiency of pLE variants.

Construct	Conjugation efficiency (C.E.)	Relative C.E. to parental bipartites
Vector	(1.3±0.1)×10^−9^	
**pLE-S3**	**(4.2±1.5)**×**10** ^−**5**^	
S3-46	(1.4±0.2)×10^−6^	0.033
S3-54	(2.6±0.4)×10^−6^	0.062
S3-104	(3.1±0.5)×10^−6^	0.074
S3-121	(3.2±1.3)×10^−6^	0.076
S3-140	(1.6±0.0)×10^−6^	0.038
S3-181	(1.6±0.2)×10^−6^	0.038
S3-418	(2.1±0.2)×10^−6^	0.050
S3-447	(4.8±0.4)×10^−7^	0.011
S3-519	(1.2±0.2)×10^−6^	0.029
S3-534	(3.8±0.4)×10^−6^	0.090
S3-542	(2.1±0.7)×10^−6^	0.050
S3-762	(5.3±1.5)×10^−9^	0.00013
S3-762+	(3.8±1.4)×10^−5^	0.905
**pLE-S10**	**(1.5±0.2)**×**10** ^−**6**^	
S10-337	(1.5±0.3)×10^−8^	0.010
S10-392	(4.5±1.1)×10^−9^	0.003
S10-418	(1.1±3.5)×10^−8^	0.007
S10-440	(8.3±4.0)×10^−9^	0.006
S10-483	(7.5±1.6)×10^−9^	0.005
S10-532	(1.1±5.0)x 10^−8^	0.007
**pLE-S4**	**(3.7±0.7)**×**10** ^−**6**^	
S4-445	(1.4±0.4)×10^−8^	0.004
S4-483	(1.2±0.3)×10^−8^	0.003
S4-532	(6.0±0.5)×10^−9^	0.002
S4-2396	(1.4±0.4)×10^−8^	0.004
S4-2404	(7.5±0.7)×10^−9^	0.002
S4-2425	(6.2±0.1)×10^−9^	0.002

pLE-S3-762 in the presence of pDE*ltrA*, + constructs harbouring bipartite introns are bolded.

### Tn5-based Genetic Screen to Uncover Functional Tripartite Ll.LtrB *trans*-splicers

We have previously used a Tn5-based genetic screen to demonstrate the bipartite *trans*-splicing versatility of the Ll.LtrB group II intron [17]. We adapted this genetic screen to investigate the tripartite *trans*-splicing potential of Ll.LtrB (Fig. 2). We subjected either the first fragment of S3 or the second fragment of the S4 or S10 bipartite introns to a Tn5 fragmentation screen, in search for functional tripartite *trans*-splicers.

The engineered Tn5 transposon harbors a *pepN* transcriptional terminator followed by the P_23_ *L. lactis* constitutive promoter (Fig. 2A) [17]. The random insertion of this transposon into a target gene severs the RNA transcript into two independent fragments: one fragment extending from the natural promoter of the gene to the transcriptional terminator in the transposon, and the second fragment extending from the P_23_ promoter in the transposon to the end of the transcript. This transposon was used to generate a saturated bank of transposon-containing pBS*ltrB* plasmids (pBS*ltrB*::Tn bank) (Fig. 2A, step 1) [17]. To generate the saturated pLE-S3::Tn bank, the PvuI/BsaI fragment of the pLE-S3 construct was replaced with the same fragment saturated with the transposon from the pBS*ltrB*::Tn bank (Fig. 2A, step 2). The subcloned fragment begins at the 3′ end of the *ltrB* exon 1 (PvuI) and spans domains I through III of the first intron fragment including a portion of the *ltrA* gene in DIV (RT domain) (BsaI) (Fig. 3A, PvuI and BsaI brackets). The pLE-S3::Tn bank consists of independent plasmids, each carrying a single Tn5 transposon between each nucleotide of the PvuI/BsaI fragment of Ll.LtrB.

The pLE-S3::Tn bank was transformed into the NZ9800Δ*ltrB L. lactis* strain and the transformants were pooled and used as donor cells for the conjugation assay (Fig. 2B). Each donor cell therefore contained an independent plasmid representing a different potential tripartite fragmentation site within the first intron piece. Since relaxase production is required to initiate the conjugative transfer of the plasmid, a pLE-S3::Tn variant could only be transferred to the recipient cell if the S3 *ltrB* transcript can tolerate fragmentation at the Tn5 insertion site, i.e. if the three Ll.LtrB fragments were able to assemble, *trans*-splice and accurately ligate the two flanking exons. Transconjugant cells would therefore harbor proficient tripartite *trans*-splicing Ll.LtrB intron variants (Fig. 2B).

Plasmids were recovered from 201 independent transconjugant cells, and the transposon insertion sites were identified by sequence analysis. The Tn5 insertions were found to be exclusively in the sense orientation according to the intron such that the terminator and promoter are functional and result in the fragmentation of the first intron piece. They were also distributed throughout the area subjected to the screen (Fig. 3A, arrowheads). Of the 201 tripartite fragmentation sites identified, 71 correspond to novel sites distributed throughout the four domains screened, most of which occur at low frequencies (black arrowheads). The remaining 130 sites have been previously observed in previous bipartite screens [17]: 32 corresponding to the full-length screens (grey arrowheads), 45 to the subdomain screens (open arrowheads), while 53 were identified in all the previous screens (checkered arrowheads). These results suggest that tripartite fragmentation throughout DI to DIV results in proficient tripartite Ll.LtrB *trans*-splicers *in vivo*.

### Analysis of Independent Tripartite *trans*-splicing Ll.LtrB Variants

Twelve tripartite introns were chosen for further investigation (Fig. 3A, asterisks). These represent fragmentation sites from each domain (DI-DIV) that were either observed in the previous bipartite screens [17], were highly represented, or novel to the tripartite screen. We first investigated the *trans*-splicing efficiencies of the chosen tripartite introns by individual conjugation assays. The rate of conjugative transfer between *L. lactis* strains is directly proportional to the splicing efficiency of Ll.LtrB from the *ltrB* transcript [16], [29]. Ten of the twelve chosen tripartite variants showed very similar conjugation efficiencies (1.2×10^−6^ to 3.8×10^−6^), only 11- to 35-fold lower than their bipartite counterpart, S3 (4.2×10^−5^) (Table 1). One isolate fragmented within DII was found to be in the 10^−7^ range (Table 1, S3-447), while the lone fragmentation site within the RT domain of LtrA allowed splicing to occur slightly above the background level of 10^−9^ (Table 1, S3-762). In accordance, Western blot analyses reveal the presence of a full-length LtrA protein for all constructs but the intron fragmented within *ltrA* (data not shown). Expression of LtrA from a second plasmid (pDE*ltrA*) led to a significant increase in *trans*-splicing efficiency, the tripartite intron being as efficient as its bipartite counterpart, S3 (Table 1, S3-762+).

**Figure 4:**
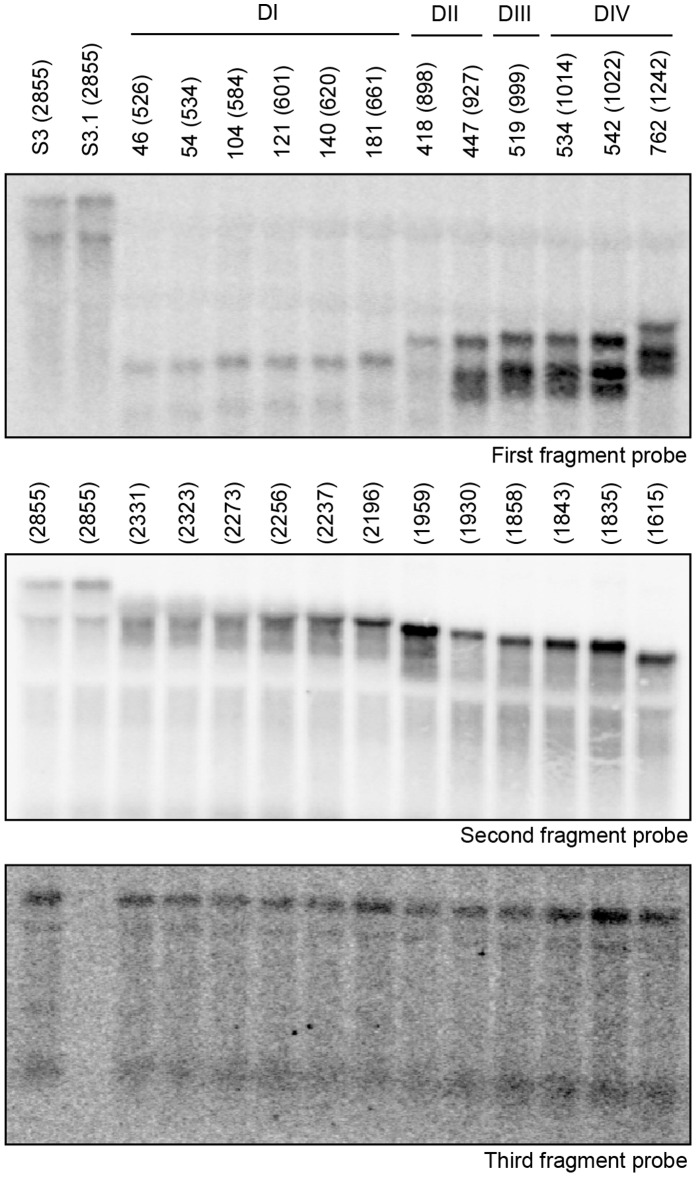
Northern blot analyses probing for the three RNA fragments of some pLE-S3::Tn tripartite introns. Northern blots were performed using total RNA from NZ9800Δ*ltrB* harbouring twelve different pLE-S3::Tn tripartite introns fragmented at positions 46 to 762. The estimated size in nucleotides of each intron fragments are shown between parentheses. The first (*A*) (*ltrB*Exon1–5′-intron until the Tn5 insertion site) and second (*B*) (Tn5 insertion site until S3) intron fragments have different but complementary sizes depending on the position of the Tn5 insertion sites while the third fragment (*C*) is invariable in size (S3–*ltrB*Exon2, ∼1400 nt). The three probes used are presented in Table 2. The two fragments from S3 are expressed from pLE-S3, S3; the first fragment from S3 is expressed from the pLE plasmid, S3.1.

We also performed Northern blots on total RNA extracts to analyze the expression and stability of the three RNA fragments involved in *trans*-splicing for the twelve isolated pLE-S3::Tn variants. Using a probe specific for each intron fragment, we detected the three intron pieces for all variants at the expected sizes (Fig. 4). We observed that the three RNA fragments seem to be expressed to similar levels, but that the stability of the first and second fragments depends on the position of the Tn5-induced fragmentation site. As expected, the sizes of the first two intron fragments are complementary and correspond to the position of the fragmentation sites while the size of the third fragment does not vary.

**Figure 5:**
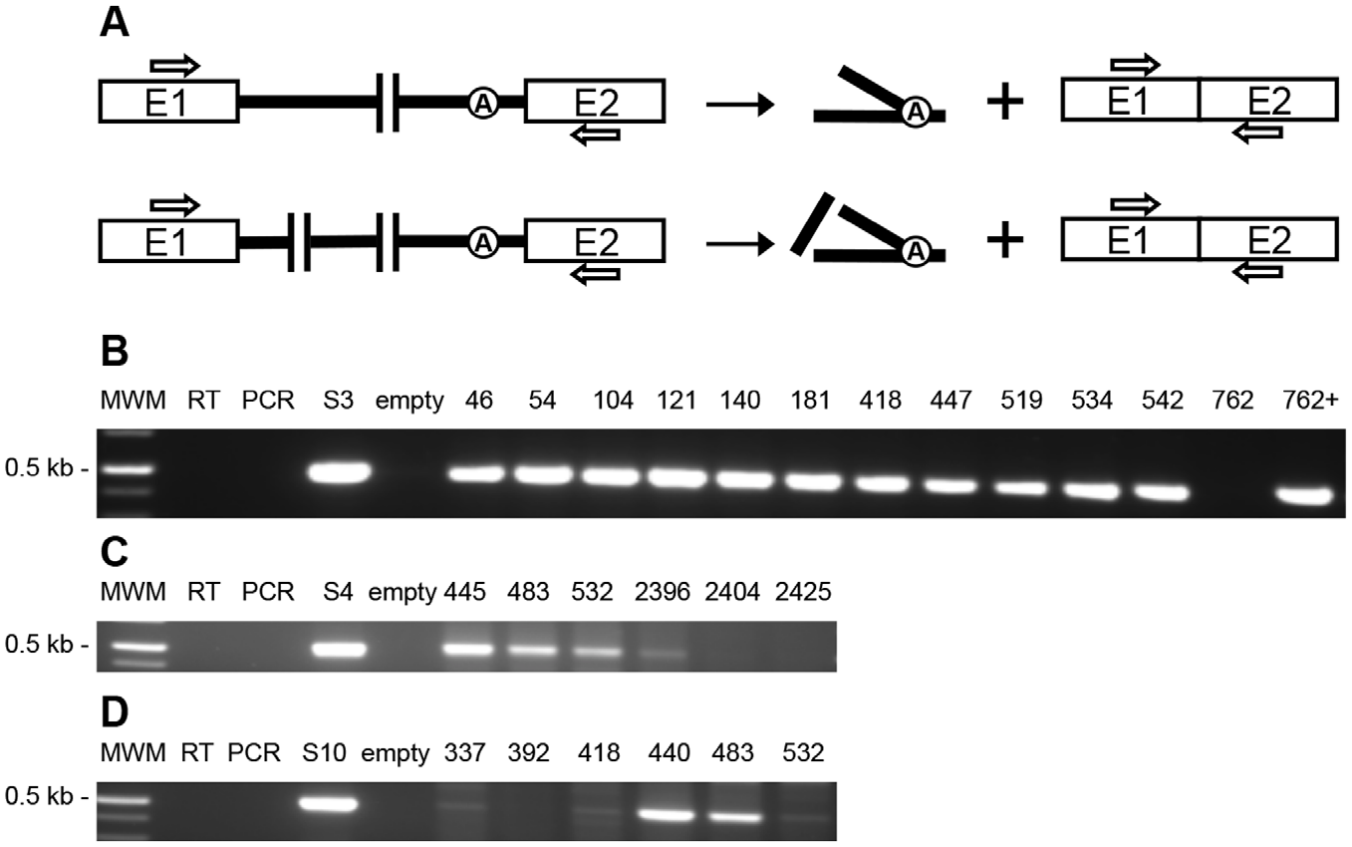
RT-PCR analysis of *ltrB* ligated exons. (*A*) The primers used to amplify the ligated exons are represented as arrows on the Ll.LtrB *trans*-splicing pathways. Amplifications were performed on total RNA extracts of NZ9800Δ*ltrB-*containing plasmids harbouring selected Ll.LtrB tripartites from S3 (*B*), S4 (*C*) and S10 (*D*). pLE-S3, S3; pLE-S4, S4; pLE-S10, S10; Molecular weight marker, MWM; control without reverse transcriptase, RT; control without polymerase, PCR; control of NZ9800Δ*ltrB* carrying the empty plasmid, empty; LtrA expressed *in trans* from pDE*ltrA*; +.

Next we monitored the presence of ligated exons in total RNA extracts from *L. lactis* donor cells harboring the various pLE-S3::Tn variants by RT-PCR. The assay was designed to generate a 521-bp fragment only if the exons are ligated following *trans*-splicing (Fig. 5A). Ligated exons were detected for all tripartite intron variants except for the S3 intron fragmented within the RT domain of LtrA (Fig. 5B). In accordance with our conjugation results, ligated exons could be detected when LtrA was provided in *trans* to complement the disruption of *ltrA* within the intron (Fig. 5B, 762+). Sequence analyses of all RT-PCR products confirmed that the exons were precisely ligated at the intron splice junctions.

These results demonstrate that the three intron fragments are produced to reasonable amounts and stable enough to assemble and fold into the catalytically active tridimensional structure leading to proper recognition of the splice junctions and accurate *trans*-splicing.

### Selection of Tripartite *trans*-splicers Starting with the S4 and S10 Bipartite Introns

Starting from a bipartite intron fragmented after the *ltrA* gene in DIV (Fig. 3, S3) we were able to select functional tripartite introns by further fragmenting the first intron fragment. We decided next to apply the same *trans*-splicing conjugation screen on the second intron fragment of a bipartite intron fragmented within DI to select for functional Ll.LtrB tripartites. In contrast to the previous screen the first fragment is invariable while the second intron fragment is the one subjected to Tn5 fragmentation.

We applied the Tn5-fragmentation screen on the second fragment of the S4 bipartrite intron fragmented at position 172 in DI (Fig. 3, pLE-S4). The pLE-S4 plasmid was shown to transfer by conjugation at a rate of 3.7×10^−6^ (Table 1). Similarly to pLE-S3::Tn, the pLE-S4::Tn plasmid bank was constructed by sub-cloning the Tn5-interrupted fragments from a pBS*ltrB*::Tn bank (Fig. 2A, step 2) (Fig. 3B, BsrGI and AatII brackets). Following the *trans*-splicing/conjugation screen only 39 transconjugants were obtained as oppose to about 2,700 for the previous screen with pLE-S3::Tn. The screen was repeated and yielded 30 transconjugants. At this point, another bipartite intron fragmented at position 44 in DI (Fig. 3, S10) was created and shown to induce conjugative transfer of its harboring plasmid pLE-S10 at a rate of 1.5×10^−6^ (Table 1). The *trans*-splicing/conjugation screen using pLE-S10::Tn was repeated 3 times and similarly to pLE-S4::Tn only generated a relatively small number of transconjugants: 81, 32 and 41.

Plasmids were recovered from 124 independent transconjugant cells from all screens (S4∶36/39, and 27/30; S10∶7/81, 14/32, and 40/41), and the transposon insertion sites were identified by sequence analysis. The Tn5 fragmentation sites were found again scattered throughout the regions subjected to the screen (Fig. 3B). Next, we investigated the *trans*-splicing efficiencies of six independent tripartite variants for both the pLE-S4::Tn and pLE-S10::Tn screens by individual conjugation assays (Table 1). All the chosen tripartite variants showed low conjugation efficiencies (4.5×10^−9^ to 1.5×10^−8^) (Table 1), 100- to 600-fold lower than their bipartite counterparts, (S4, 3.7×10^−6^; S10, 1.5×10^−6^) and only 3.5- to 11.5-fold over background (Vector, 1.3×10^−9^). However, even though the conjugation efficiencies of these tripartite variants were significantly lower compared to their bipartite counterparts we were able to detect ligated exons by RT-PCR for the majority of them (Fig. 5C, D).

## Discussion

Using *trans*-splicing conjugation assays we previously demonstrated the bipartite *trans*-splicing versatility of the Ll.LtrB group II intron. Ll.LtrB was shown to support fragmentation at natural fragmentation sites [16] and at several new positions throughout its structure [17]. We also assessed the contribution of base-pairing interactions between intron fragments during *trans*-splicing of various bipartite introns *in vivo* [28].

While several bipartite group II introns were previously described, only two tripartite group II introns, interrupting the chloroplast *psaA* gene of *C. reinhardtii* [21] and the mitochondrial *nad5* gene of *O. berteriana* [22], are currently documented. In this study, we adapted our Tn5-based genetic screen to explore the *trans*-splicing ability of Ll.LtrB when fragmented into three pieces. The power and reliability of this experimental approach designed to select fragmented introns that can assemble and splice *in trans* from a bank of fragmented introns was previously demonstrated [17]. Tripartite *trans*-splicing variants were selected from bipartite introns using sensitive *trans*-splicing/conjugation assays in *L. lactis* (10^3^- to 10^4^-fold, Table 1) (Fig. 2B). Through a Tn5-based genetic screen we isolated a series of tripartite *trans*-splicing Ll.LtrB variants found to be fragmented throughout the stems and loops of DI through DV (Fig. 3). The best way to fully address the *trans*-splicing potential of Ll.LtrB in three pieces would have been to assess all potential combinations of two fragmentation sites throughout the intron. Since it was statistically impossible to generate two successive saturated bank of fragmentation within Ll.LtrB, we applied our fragmentation screen on the first or the second fragment of specific bipartite introns (Fig. 3, S3, S4 and S10).

We first selected for active tripartite introns fragmented in the first piece of the S3 Ll.LtrB bipartite intron. A large proportion of the tripartite introns isolated in this screen where fragmented at sites previously observed in bipartite screens [17]. Nevertheless, 35% of the fragmentation sites identified were exclusive to this screen (71/201). The analysis of twelve selected tripartite introns showed that they *trans*-splice only 11- to 35-fold less efficiently than their bipartite counterpart S3. In comparison, S3 was shown to splice *in trans* only 4.8-fold less efficiently than the *cis*-splicing intron [16]. Ligated exons were amplified by RT-PCR and the sequence showed precise joining of the flanking exons corroborating the accurate *trans*-splicing of the tripartite introns at the molecular level.

The lack of fragmentation observed within DIV confirms the importance of LtrA as a co-factor for Ll.LtrB *trans*-splicing [16], [17]. DIV harbours the *ltrA* gene and the primary binding site for LtrA whose maturase activity is essential for efficient splicing *in vivo*. Only one tripartite intron variant was found to be severed within the *ltrA* gene at position 762 (RT domain). Although LtrA is not detectable by Western blot, six potential start codons downstream of the fragmentation site are found to be in the correct reading frame. The use of one of these initiation codons may lead to low levels of expression of a N-terminal truncated LtrA protein with maturase activity. Accordingly, the expression of LtrA *in trans* from a second plasmid rescued splicing of this tripartite intron inducing conjugation efficiencies at levels similar to the bipartite S3 intron. This shows that the additional fragmentation site within DIV does not significantly affect Ll.LtrB *trans*-splicing efficiency. This is reminiscent of the previously selected bipartite introns fragmented within *ltrA* but downstream from the maturase domain. We proposed that these introns expressed C-terminal truncated versions of active maturases. Similarly, the expression of full-length LtrA from a second plasmid significantly increased the *trans*-splicing efficiency of these introns [17].

As previously observed in our screens for bipartite introns [17], fragmentation is not tolerated in functionally important regions of the intron including EBS1, EBS2, DVI and the splice sites. In addition, we do not observe Tn5 insertion sites within the inter-domain nucleotides of the central wheel, consistent with their important role in splicing [4]. This is also generally true for the structurally important long-range tertiary interactions (Figure 3, pairs of Greek letters), with some exceptions in the α-α’, β-β’, κ-κ’, and ζ-ζ’ interactions. The α-α’ and β-β’ long-range interactions are involved in bringing the two halves of DI (Ia,b,c and Id) together as the intron folds into its active tertiary structure. The folding of DI is referred to as the rate-limiting step as this domain serves as the scaffold for the folding of the remaining domains. However, the α-α’ and β-β’ interactions seem to occur only following the partial docking of the catalytic domain, DV [5]. Therefore, these interactions may not be as important for the initial folding of the individual domains and the assembly of the intron fragments, perhaps making these regions more tolerable to fragmentation. Alternative explanations may also rationalize the tolerance of some motifs involved in long-range tertiary interactions to fragmentation. Since the Tn5 transposon creates a direct repeat upon insertion, causing the second intron fragment to start 9 nt upstream from the fragmentation site, this may allow the production of intact and functional motifs that can still interact with their partners. On the other hand, some of these disrupted tertiary contacts may indeed be non-functional but redundant, still leading to some level of proper intron folding.

Through Northern blots we clearly detected the three RNA fragments of the twelve Ll.LtrB tripartite variants chosen. All three fragments seem to be expressed to similar levels while some degradation products can be seen. The third fragment appears to be the least stable when compared to the first two fragments. In identifying the first intron fragment of the tripartite variants, we observe an increase in the intensity of the bands when fragmentations occur downstream of DI. Since DI serves as a docking site for the remaining domains, its folding as a complete module possibly contribute to the increase in stability of the fragments extending into DII to DIV. Even though certain tripartite variants appear to have fragments more stable than others this does not seem to significantly affect their *trans*-splicing efficiencies. Although we hypothesize that the location of the fragmentation site determines the assembly and *trans*-splicing efficiency, the stability of the first two fragments may not be a significant factor, especially if the third fragment is available in limiting amounts. Nevertheless, the three RNA fragments retain the resilience to fold, assemble and *trans*-splice accurately *in vivo*.

In a complementary approach to fragmenting the first piece of a bipartite intron (S3), we submitted the second fragment of bipartite introns severed in DI at either position 172 (S4) or 44 (S10) to our fragmentation screen. We first noticed that the S4 and S10 bipartite introns were not as proficient as S3, *trans*-splicing respectively 11- and 28-fold less efficiently. In addition, significantly less transconjugants were obtained following the *trans*-splicing conjugation screens. Accordingly, the analysis of independent transconjugants from the pLES4::Tn and pLES10::Tn screens showed conjugation efficiencies 100- to 600-fold lower than their bipartite counterparts only 11.5- to 3.5-fold over background. This is in contrast to the pLES3::Tn transconjugants that *trans*-splice only 11- to 35-fold less efficiently than S3 and 2,900- to 920-fold over background. Similarly to what was previously observed following bipartite screens, when the end of DIV was subjected to transposon insertion (S4), introns fragmented within the *ltrA* gene downstream of the maturase domain were selected suggesting the expression of truncated LtrA proteins with maturase activity [17]. Our data thus imply that it is easier to further fragment a bipartite intron already severed in DIV than in DI. This may be due to the fact that DI is the scaffold for tertiary folding of group II introns. Nevertheless, numerous active tripartite introns fragmented in the vicinity of S4 and S10, and elsewhere within DI, were selected starting from the S3 bipartite intron. At the same time, the only two natural *trans*-splicing introns fragmented in DI are tripartite introns also fragmented in DIV. While the great majority of bipartite introns were found fragmented in DIV, no bipartite introns were ever found fragmented in DI. Taken together, this suggests that the two natural tripartite introns probably underwent fragmentation in DIV before being further fragmented in DI.

The discovery of naturally occurring group II introns fragmented into two or three pieces supports the theories proposing that self-splicing group II introns are the progenitors of nuclear introns and that the spliceosomal snRNAs were derived from fragments of group II introns [11]. Sharp proposed that the two natural tripartite group II introns interrupting the chloroplast *psaA* gene of *C. reinhardtii* [21] and the mitochondrial *nad5* gene of *O. berteriana* [22] represent intermediate fragmentation steps in the evolution of a group II intron towards the five snRNAs [11]. Here, we show that the Ll.LtrB group II intron from *L. lactis* is quite tolerant to tripartite fragmentation as it can be fragmented into three pieces not only in DI and DIV like the two organellar tripartite introns but at various locations throughout its structure. The selected Ll.LtrB tripartite introns retained the ability to fold correctly, assemble and accurately splice in *trans*. This suggests that following fragmentation, *trans*-splicing group II introns would be under significant pressure to improve their splicing efficiency in order to restore the expression level of the gene they interrupt. Such adaptation would lead to fragmented introns that *trans*-splice more efficiently and that can be further fragmented. Since no functional bipartite or tripartite group II introns were found yet in bacterial genomes the selected introns represent the first evidence of functional tripartite group II introns in bacteria. Assembly of three intron RNA fragments during *trans*-splicing *in vivo* probably involves a complex combination of base pairing, long-range tertiary interactions and the potential involvement of the splicing co-factor LtrA [16], [17], [28].

Demonstration that a bacterial group II intron can be fragmented in three pieces at multiple sites throughout its structure and still be functional provides additional experimental support to the theory proposing that the five snRNAs of the eukaryotic spliceosome were derived from group II intron fragments.

## Materials and Methods

### Bacterial Strains and Plasmids Used in this Study

The *Escherichia coli* DH10β strain was used for both cloning and plasmid amplification, and was grown in LB broth at 37°C with shaking. The methylase-free *E. coli* GM119 strain was used to isolate non-methylated DNA since methyl-free PvuI digested DNA fragments are ligated more efficiently. *L. lactis* strains NZ9800Δ*ltrB*::tet (NZΔL-b) (Tet^R^) [30] and LM0231 (Fus^R^) [16] were used as donor and recipient strains for conjugation, respectively. They were grown in M17 broth supplemented with 0.5% glucose (GM17) at 30°C without shaking. The following antibiotic concentrations were used: chloramphenicol (Cam), 10 µg/mL; fusidic acid (Fus), 25 µg/mL; spectinomycin (Spc), 300 µg/mL; erythromycin (Erm), 300 µg/mL.

The pLE-S3, pLE-S4 and pLE-S10 constructs were engineered and used to generate the pLE-S3::Tn, pLE-S4::Tn and pLE-S10::Tn variants (Fig. 2A). The origin of transfer (*oriT*) from *L. lactis* SF was PCR-amplified (Table 2, primers) and cloned into the unique PstI site of the pLE1 plasmid [31]. Next, the multiple cloning site (MCS) from pDL278 previously engineered to contain two divergent constitutive promoters, P_23_ left and P_23_ right [16], was excised with SalI, blunted and cloned into the unique SmaI site of pLE1. Before its transfer from pDL278 to pLE1, two PvuI and three BsaI sites were destroyed from the MCS using the QuikChange® multi site-directed mutagenesis kit (Stratagene), to facilitate downstream cloning (Table 2, primers). The *ltrB* gene was inserted as two pieces fragmented at position S3, S4 or S10 within Ll.LtrB: the first piece (*ltrB*Exon1–5′-intron) under the control of P_23_ right (BssHII) and the second piece (3′-intron–*ltrB*Exon2) under the control of P_23_ left (NotI). These constructs were shown to be mobilizable by conjugation (Table 1). pDE*ltrA* was previously described [17].

**Table 2:** Primers.

Name	Sequence 5′ –3′
5′-*oriT* (PCR)	ACGTCTGCAGATCCATGGATGACCTCGAAAAACGAGAGGG
3′-*oriT* (PCR)	ACGTCTGCAGGGAAGAGTTCCTACGCATTTGG
Mutant PvuI 1	TGGGAAGGGCGACCGGCGCGCAATTC
Mutant PvuI 2	TTGTCATCACGACCGGTGCGGGCCTC
Mutant BsaI 1	AGCCTCTCAGAGGTTTCAGAATCGCCAGG
Mutant BsaI 2	CTCGCATGGGGAAACCCCACACTACC
Mutant BsaI 3	CTCGCATGGGGAAACCCCACACTACC
First piece intron probe	GACACTAGTTTTCGCGATTATTATAGACTTAACACCCTATCTGGGC
Second piece intron probe	TTAGACAGCTGTATTCCATAAG
Third piece intron probe	CCGCCTTGTTCACATTACTGTGAC

CTGCAG: PstI, CGATCG: PvuI, CGACCG: PvuI destroyed, GGTCTC: BsaI, GGTTTC and GAAACC: BsaI destroyed.

The Tn5 transposon variant used in this study was previously generated [17]. The pMOD™-2<MCS> Transposon Construction Vector (Epicentre) contains the *pepN* transcriptional terminator, a gene conferring spectinomycin (Spc) resistance, and the P_23_ constitutive promoter (Fig. 2A). With this randomly inserting transposon, a saturated pBS*ltrB*::Tn bank was previously created such that a single Tn5 insertion occurred between each nucleotide of the plasmid; every possible Tn5 insertion was represented 22 times [17]. To create a saturated Tn5 bank of the first piece of the S3 intron, the PvuI/BsaI fragment of the pLE-S3 construct was replaced with the same fragment from the pBS*ltrB*::Tn bank. The pLE-S3::Tn bank generated was represented 4.9 times in DH10β ElectroMAX cells (Invitrogen) and 2.8 times in NZ9800Δ*ltrB* following transformation. The Tn5 transposon was therefore inserted between each nucleotide of the PvuI/BsaI fragment of the intron 2.8 times immediately preceding the *trans*-splicing/conjugation assay. The same procedure was done to subject the second intron fragment to the screen in pLE-S4 (BsrGI/AatII) and pLE-S10 (EagI/BsaI). The statistical representation of the generated banks was calculated by dividing the number of Cam/Spc colonies recuperated following transformation of the Tn5-containing plasmids by twice the size of the fragments (PvuI/BsaI, 2×885 bp; BsrGI/AatII, 2×2278 bp; EagI/BsaI, 2×798 bp). This method of calculation takes into consideration that Tn5 can insert randomly in both orientation within the target plasmid [32], [33].

### Conjugation Assays

Conjugations were performed on milk plates as previously described [16]. Conjugation efficiencies of the mobilizable plasmids were calculated as the ratio of transconjugant cells (Cam/Fus) to donor cells (Cam) for three independent assays.

### Northern Blot Hybridizations and RT-PCR

Total RNA was isolated from NZ9800Δ*ltrB* cells harbouring various selected tripartite introns as previously described [16]. For Northern blot hybridization, 10 µg of total RNA was resolved on 1% agarose gel containing 5% formaldehyde and transferred by capillarity to a nylon membrane. The membranes were hybridized with the appropriate 5′-^32^P labelled oligonucleotide probe (Table 2) and visualized with the Molecular Imager FX software (Bio-Rad). RT-PCR assays were performed as previously described [16].
